# Physiological and pathological phosphorylation of tau by Cdk5

**DOI:** 10.3389/fnmol.2014.00065

**Published:** 2014-07-15

**Authors:** Taeko Kimura, Koichi Ishiguro, Shin-ichi Hisanaga

**Affiliations:** ^1^Laboratory of Molecular Neuroscience, Department of Biological Sciences, Tokyo Metropolitan UniversityHachioji, Japan; ^2^Department of Neurology, Graduate School of Medicine, Juntendo UniversityBunkyo, Japan

**Keywords:** Cdk5, p25, p35, tau, Alzheimer’s disease, FTDP-17, tauopathy, phosphorylation

## Abstract

Hyperphosphorylation of microtubule-associated protein tau is one of the major pathological events in Alzheimer’s disease (AD) and other related neurodegenerative diseases, including frontotemporal dementia with parkinsonism linked to chromosome 17 (FTDP-17). Mutations in the tau gene *MAPT* are a cause of FTDP-17, and the mutated tau proteins are hyperphosphorylated in patient brains. Thus, it is important to determine the molecular mechanism of hyperphosphorylation of tau to understand the pathology of these diseases collectively called tauopathy. Tau is phosphorylated at many sites via several protein kinases, and a characteristic is phosphorylation at Ser/Thr residues in Ser/Thr-Pro sequences, which are targeted by proline-directed protein kinases such as ERK, GSK3β, and Cdk5. Among these kinases, Cdk5 is particularly interesting because it could be abnormally activated in AD. Cdk5 is a member of the cyclin-dependent kinases (Cdks), but in contrast to the major Cdks, which promote cell cycle progression in proliferating cells, Cdk5 is activated in post-mitotic neurons via the neuron-specific activator p35. Cdk5-p35 plays a critical role in brain development and physiological synaptic activity. In contrast, in disease brains, Cdk5 is thought to be hyperactivated by p25, which is the N-terminal truncated form of p35 and is generated by cleavage with calpain. Several reports have indicated that tau is hyperphosphorylated by Cdk5-p25. However, normal and abnormal phosphorylation of tau by Cdk5 is still not completely understood. In this article, we summarize the physiological and pathological phosphorylation of tau via Cdk5.

## INTRODUCTION

Alzheimer’s disease (AD) is the most common neurodegenerative dementia and affects more than 35 million people worldwide. Thus, the development of therapeutic methods is urgently needed to determine the underlying molecular mechanism of AD. Major pathological hallmarks of AD include senile plaques and neurofibrillary tangles (NFT), which consist mainly of amyloid β peptide (Aβ) and hyperphosphorylated tau, respectively (Mattson, 1997; Bettens et al., 2010). Mutations of the amyloid precursor protein (APP) and presenilin, a component of γ-secretase, are found in familial AD, and previous studies have established the hypothesis of the amyloid cascade (Huse and Doms, 2000; Gotz et al., 2004; Hardy, 2006; Bertram et al., 2010). On the basis of this hypothesis, great effort has been paid to develop drugs to reduce Aβ production or to clear Aβ, but successful results have not yet been obtained. In contrast, it has been shown that tau pathology is more closely related to neuronal loss (Gómez-Isla et al., 1997; Ingelsson et al., 2004). Tau is a genetic factor of a neurodegenerative disease known as frontotemporal dementia parkinsonism linked with chromosome 17 (FTDP-17; Hutton et al., 1998; Poorkaj et al., 1998; Spillantini et al., 1998). FTDP-17 tau mutants are highly phosphorylated in patient brains. Regardless of whether phosphorylation is a cause of FTDP-17, it is still critical to determine the neuronal milieu in which tau hyperphosphorylation occurs. Cyclin-dependent kinase 5 (Cdk5) is a major tau kinase that is involved in abnormal phosphorylation in AD brains (Imahori and Uchida, 1997; Cruz and Tsai, 2004; Engmann and Giese, 2009). Here, we summarize the phosphorylation of tau by Cdk5. To the best of our knowledge, this is the first review article focused specifically on Cdk5 phosphorylation of tau.

## Tau PROTEIN

**FIGURE 1:**
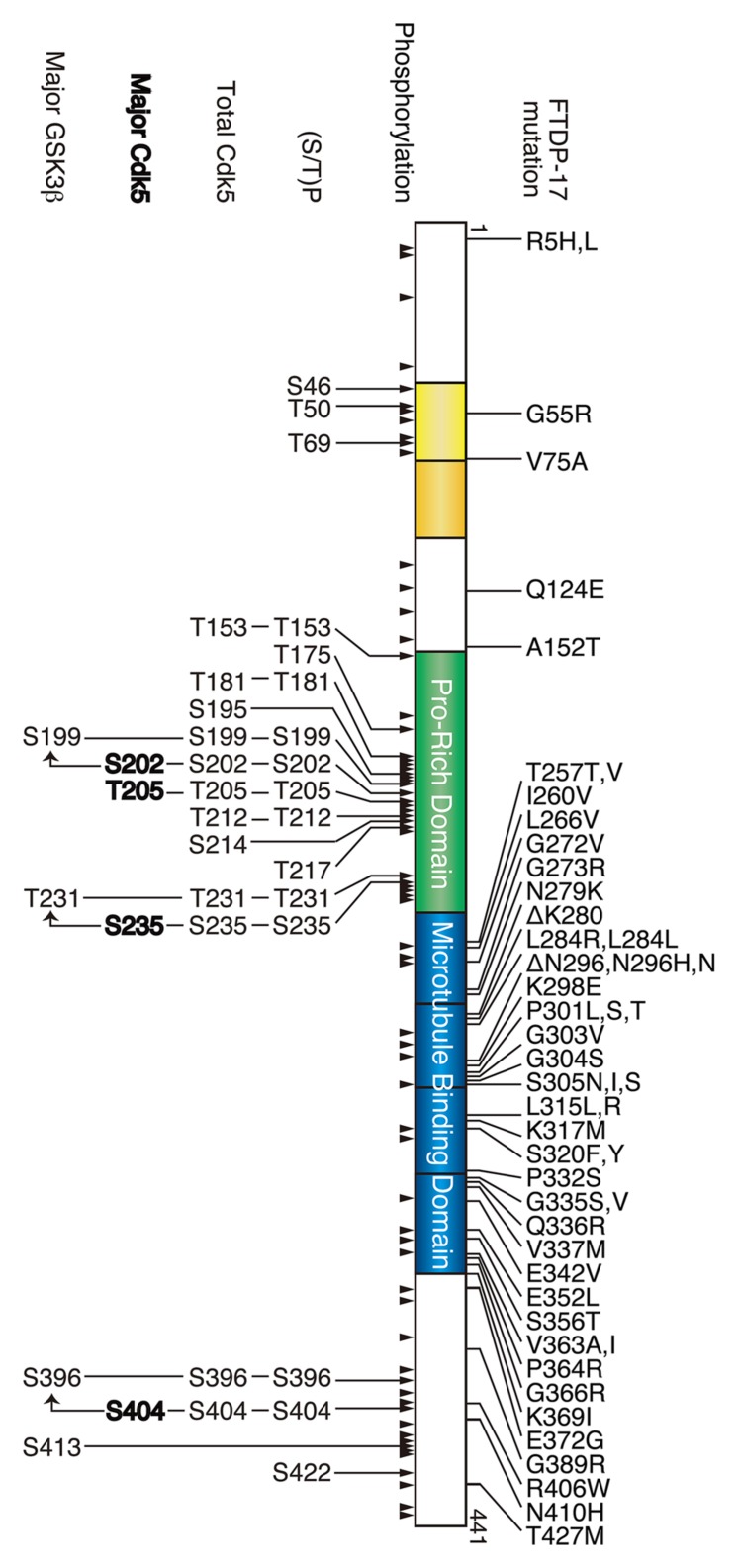
**Frontotemporal dementia with parkinsonism linked to chromosome 17 (FTDP-17) mutation and phosphorylation sites in tau.** The longest human tau consists of 441 amino acids. Two N-terminal insertions are labeled in yellow, the proline-rich region is labeled in green, and four microtubule-binding repeats are shown in blue. FTDP-17 mutations are shown above and the phosphorylation sites are shown below tau. All phosphorylation sites reported thus far are indicated by arrowheads, and the (Ser/Thr)Pro sequences, all Cdk5 phosphorylation sites, major Cdk5 phosphorylation sites and major GSK3β phosphorylation sites are shown in the upper to lower row.

Tau is a member of the heat-stable microtubule-associated proteins (MAPs), which consist of MAP2 and MAP4 (Dehmelt and Halpain, 2005). Tau, as well as MAP2, is mainly expressed in mammalian neurons. While MAP2 is localized in dendrites, tau binds to microtubules that are present in axons and is thus often used as an axonal marker (Binder et al., 1985). Similar to MAP2, tau binds to microtubules via the C-terminal microtubule-binding repeats, which consist of three or four imperfect repeats of 31 or 32 amino acids. There are six isoforms in human tau (Goedert et al., 1989) that are dependent on the presence or absence of one or two N-terminal insertions and a C-terminal region with three (3R) or four (4R) microtubule-binding repeats. The longest isoform of human tau is composed of 441 amino acids; the phosphorylation sites and mutation sites are usually numbered according to this isoform of tau (**Figure 1**). We also used this notation in this article. Physiologically, tau promotes microtubule assembly and stabilizes microtubules by laterally binding to the surface of microtubules (Matus, 1994; Mandelkow et al., 1995). In addition to the classical functions, new functions in signaling and cytoskeletal organization have emerged (Morris et al., 2011). These activities are regulated by phosphorylation in the microtubule-binding repeat or the franking region by a number of protein kinases (Imahori and Uchida, 1997; Johnson and Stoothoff, 2004; Gong and Iqbal, 2008; Avila et al., 2010). Thus, the physiological function of tau is regulated by phosphorylation. Tau is a naturally unfolded protein with an extended structure but aggregates into NFTs in the brains of AD patients. A number of neurodegenerative diseases with tau aggregates are collectively known as tauopathy (Iqbal et al., 2005; Spillantini and Goedert, 2013). Tau in aggregates is hyperphosphorylated, and this hyperphosphorylation is a feature employed for the diagnosis of diseases. However, it is not completely known how pathological tau is hyperphosphorylated and what role hyperphosphorylation plays in aggregate formation and disease development.

## Cdk5 AS A tau PROTEIN KINASE

Cdk5 was first purified as one (TPKII) of two tau protein kinases, namely TPKI and TPKII, from a bovine brain microtubule fraction (Ishiguro et al., 1992), as neuronal cdc2-like kinase (nclk) from bovine brain extracts (Lew et al., 1992), and as a KSP sequence phosphorylating kinase from rat spinal cord (Shetty et al., 1993). Purified Cdk5 is a complex that consists of the Cdk5 catalytic subunit with a molecular mass of approximately 31 kDa and a polypeptide with a molecular mass of approximately 23–25 kDa (p25). p25 was subsequently shown to be an N-terminal truncated form of its full-length version of p35 (Lew et al., 1994; Tsai et al., 1994; Uchida et al., 1994). Cdk5 has attracted attention as a potential disease tau kinase because Cdk5 phosphorylated tau at sites that were hyperphosphorylated in AD brains (Imahori and Uchida, 1997). Importantly, there is an accumulation of p25 in AD brains with higher phosphorylation ability of Cdk5-p25 for tau compared Cdk5 activated by p35 (Patrick et al., 1999), which highlights the importance of Cdk5-p25 in abnormal tau phosphorylation.

## GENERAL PROPERTIES OF Cdk5

**FIGURE 2:**
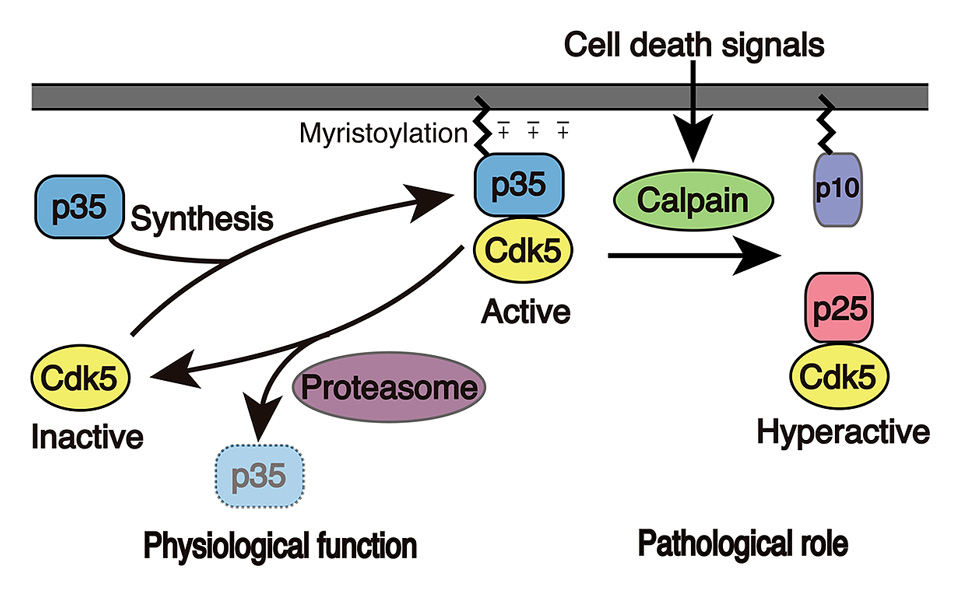
**Activation mechanism of Cdk5.** Cdk5 alone is an inactive catalytic subunit. Cdk5 is activated by the p35 Cdk5 activator and is concomitantly recruited to membranes because p35 associates with membranes via myristoylation of its N-terminal region. p35 is a protein with a short-life span and is proteasomally degraded. When neurons suffer from stress or encounter death signals, calpain is activated and cleaves p35 into a p25 C-terminal fragment. p25 has a longer half-life and is dissociated from membranes, where it is able to phosphorylate additional proteins.

Cdk5 is a member of the Ser/Thr cyclin-dependent kinases (Cdks). Cdk5 is a catalytic subunit and is activated by binding to its regulatory subunit, p35 or p39 (**Figure 2**). Cdk5 has a 55~60% amino acid sequence homology to well-known cell cycle Cdks (Hellmich et al., 1992; Meyerson et al., 1992), such as Cdk1, 2, 4, and 6, but its activators, namely p35 and p39, display no homology to cyclins, which are activators of cell cycle Cdks. p35 and p39 consist of 307 and 369 amino acids, respectively, with a Cdk5 activation domain in the C-terminal region (Lew et al., 1994; Tsai et al., 1994; Uchida et al., 1994; Tang et al., 1995; Zheng et al., 1998). The crystal structure has revealed that the activation domain of p35 has a tertiary structure, which resembles cyclin A in the Cdk2-cyclin A complex, that explains its ability to activate Cdk5 (Tarricone et al., 2001). Although cell cycle Cdks are activated at a particular cell cycle phase in proliferating cells and promote cell cycle progression, Cdk5 is mainly active in post-mitotic neurons, although Cdk5 is expressed widely in many cells types and tissues. This is because p35 and p39 are predominantly expressed in neurons (Lew et al., 1994; Tsai et al., 1994; Uchida et al., 1994; Tang et al., 1995; Zheng et al., 1998). The number of reports describing the kinase activity of Cdk5 in extra-neuronal cells or tissues is increasing (Rosales and Lee, 2006), but the activation mechanism and function are poorly understood yet. While p35 could be the major activator, cyclin I was indicated to be an activator of Cdk5 in kidney podocytes (Brinkkoetter et al., 2009).

Cdks are a family of proline-directed protein kinases (PDPKs) that phosphorylate Ser or Thr residues followed by proline, that is, Ser/Thr-Pro sequences. The substrate specificity of Cdk5 is very similar to that of Cdk1-cyclin B (Hisanaga et al., 1995). There are at least three different PDPKs, namely MAP kinases such as ERK1/2, GSK3, and Cdks, but their substrate preferences are slightly different. Cdk5 prefers the (Ser/Thr)-Pro motif with a basic amino acid at the second C-terminal site, (Ser/Thr)-Pro-X-(Lys/Arg) (X is any amino acid; Hisanaga et al., 1991, 1993; Beaudette et al., 1993; Sharma et al., 1999). ERK1/2 phosphorylates Pro-X-(Ser/Thr)-Pro sequences (Davis, 1993), and GSK3β can phosphorylate (Ser/Thr)-(X)_3_-(pSer/pThr) with priming phosphorylation at +4 site in addition to (Ser/Thr)Pro sequences (Cohen and Frame, 2001).

In contrast to cell cycle Cdks, Cdk5 does not require phosphorylation of the Cdk5 subunit in the activation loop for activation. Binding of the regulatory subunits p35 and p39 is sufficient for activation (Qi et al., 1995; Lee et al., 1997; Hisanaga and Endo, 2010). The kinase activity of Cdk5 is mainly determined by the available protein amounts of p35 or p39 in neurons. p35 and p39 are proteins with a short life in which the half-life is about 30 min and 120 min for p35 and p39, respectively (Patrick et al., 1998; Saito et al., 1998; Patzke and Tsai, 2002; Minegishi et al., 2010). p35 and p39 are degraded by the ubiquitin-proteasome system as are other short half-life proteins. Thus, one major factor that regulates Cdk5 activity is degradation of p35 and p39 via proteasomal degradation. Phosphorylation at Thr138 in p35 by Cdk5 stimulates this degradation (Kamei et al., 2007). Because phosphorylation decreases with aging, the half-life of p35 becomes longer in adult mouse brains; however, it is not known how the phosphorylation state is determined or the identity of the E3 ubiquitin ligase for p35.

The cellular localization is also different between cell cycle Cdks and Cdk5. While cell cycle Cdks are mainly present in the nucleus, where they promote the cell cycle, active Cdk5 associates with membranes in the cytoplasm. This is achieved by the membrane binding properties of p35 and p39, which are myristoylated at the N-terminal Gly (**Figure 2**; Patrick et al., 1999; Patzke and Tsai, 2002; Asada et al., 2008, 2012). There are excess amounts of Cdk5 compared to p35 and p39, and these free Cdk5 molecules are soluble. The binding of p35 or p39 not only activates Cdk5, but it also induces its membrane association. Membrane association is also supported by Lys residues in the N-terminal p10 region of p35 and p39 (+charge in p35 in **Figure 2**). This membrane association may not only restrict its targets to proteins localized to the submembranous regions but also prohibits its nuclear translocation.

When neurons suffer from stress, death signals or overexcitation, large influxes of Ca^2+^ enter into the cytoplasm, resulting in the activation of the calcium-dependent protease calpain (Ono and Sorimachi, 2012). Calpain cleaves p35 to p25, an N-terminal truncated form that consists of the C-terminal Cdk5 activation domain (**Figure 2**; Patrick et al., 1999; Kusakawa et al., 2000; Lee et al., 2000). Subsequently, in contrast to Cdk5-p35, Cdk5-p25 is released from the membranes and is capable of accessing proteins. In addition, a component of Cdk5-p25 is known to enter into the nucleus to activate the cell cycle machinery. Furthermore, Cdk5-p25 acquires a longer half-life, resulting in the net activation of Cdk5 (Patrick et al., 1999; Minegishi et al., 2010).

## Cdk5 PHOSPHORYLATION SITES IN tau

Tau is phosphorylated at approximately 45 sites in AD brains (**Figure 1**). Phosphorylation characteristically occurs at many (Ser/Thr)-Pro sequences (**Figure 1**; Hasegawa et al., 1992; Morishima-Kawashima et al., 1995). Among the 16 (Ser/Thr)-Pro sequences in tau, Cdk5 phosphorylates 9–13 sites (Chauhan et al., 2005; Hanger et al., 2009). However, the reported sites are not always the same. Initially, Ishiguro’s group identified Ser202, Thr205, Ser235, and Ser404 as TPKII (Cdk5-p25) phosphorylation sites using amino acid sequencing (Arioka et al., 1993). In addition, using purified nclk (Cdk5-p25), Paudel et al. (1993) reported Ser195, Ser202, Thr205, Thr231, Ser235, Ser396, and Ser404. Illenberger et al. (1998) demonstrated Ser202, Thr205, Ser235, and Ser404 to be major sites with Thr153 and Thr212 as minor sites in *in vitro* Cdk5-p25 phosphorylated tau using 2D-phospho-peptide mapping and mass spectrometry analysis. Phosphorylation sites of human tau via *in vitro* recombinant Cdk5-p20 (shorter activation construct than p25) include Thr181, Thr205, Thr212, Thr217, Ser396, and Ser404, which were determined using mass spectrometry (Lund et al., 2001). Liu et al. (2002) reported that Cdk5-p25 phosphorylates Tau at Thr181, Ser199, Ser202, Thr205, Thr212, Ser214, Ser217, Thr231, Ser235, Ser396, and Ser404 using a repertoire of phospho-specific antibodies. Using NMR, Landrieu et al. (2010, 2011) analyze tau phosphorylation by Cdk2-cyclin A3 and Cdk5-p25; while Cdk2-cyclin A3 phosphorylated Thr153, Ser199, Ser202, Thr205, Thr231, Ser235, and Ser404, with high levels of phosphate incorporation at Ser202/Thr205 and Thr231/Ser235, Cdk5-p25 needed GSK3β for the same phosphorylation profile with Cdk5-p25 providing Ser202, Thr205, Ser235, and Ser404 as major sites. We believe that these differences in phosphorylation sites were due to the methods and kinase preparations used for analysis. The use of phospho-specific antibodies may detect minor phosphorylation sites, which are sometimes below the detection level of biochemical methods. Further, immunoblotting with phospho-specific antibodies has a problem in quantification of multiply phosphorylated proteins (Prabakaran et al., 2011). Purification or *in vitro* reconstruction of active Cdk5 is also challenging. If the kinase activity is not sufficiently high, then the contribution of contaminating kinases may present challenges.

**FIGURE 3:**
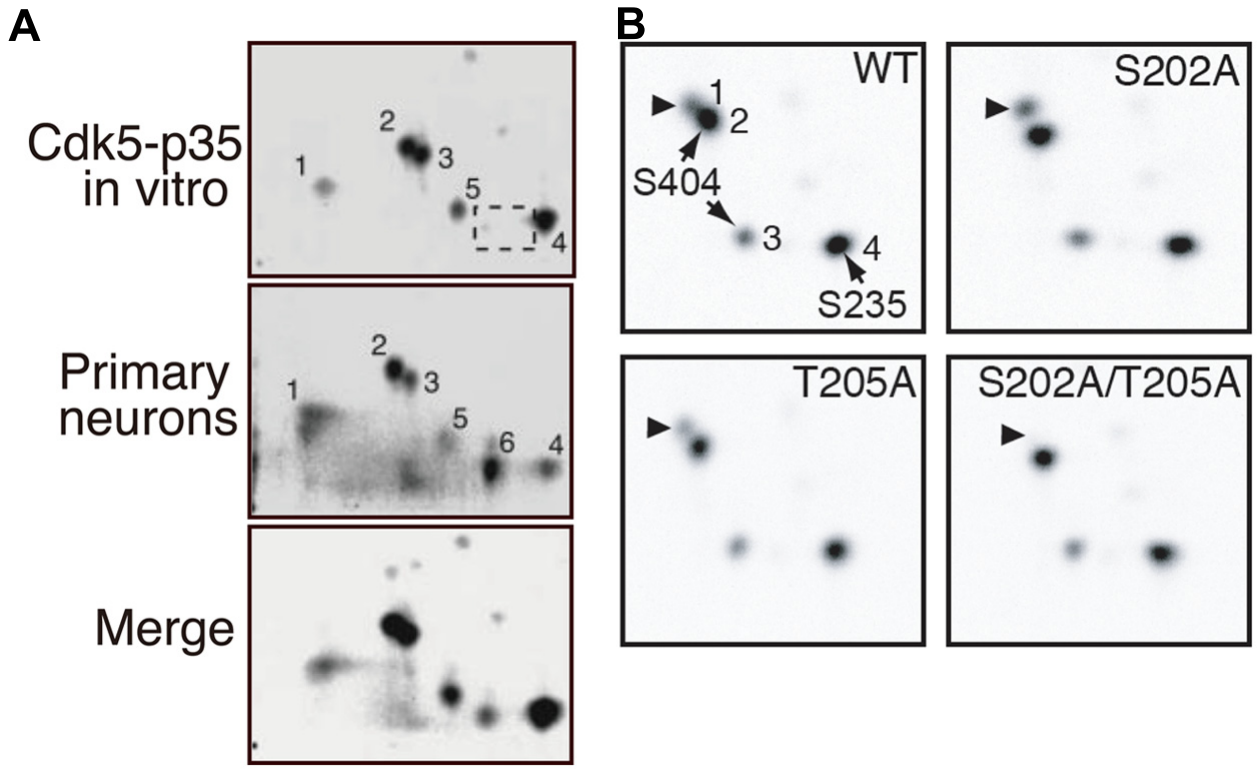
**2D-phosphopeptide mapping of tau phosphorylation by Cdk5. (A)** Comparison of phosphorylation spots between tau phosphorylated *in vitro* via Cdk5-p35 (upper) and in cultured neurons (middle). The lower panel is a merged image. Spot 1 is doubly phosphorylated at Ser202 and Thr205. Spot 2 is phosphorylated at Ser202 or Thr205, spots 3 and 5 at Ser404 and spot 4 at Ser235. Spot 6, which was found in rat cortical neurons **(B)**, was missing in tau phosphorylated by Cdk5-p35 *in vitro* (dotted square). The spot was not detected in T181A mutant (Sakaue et al., 2005). Reproduced from Sakaue et al. (2005). **(B)** 2D-phosphopeptide map of tau WT (upper left), tau S202A (upper right), tau T205A (lower left), and tau S202A/T205A. Spot 1 corresponds to spot 2 of **(A)**, spot 2 to spot 3 of **(A)**, and spot 3 to spot 5 of **(A)**. Reproduced from Kimura et al. (2013).

We have determined the phosphorylation sites of tau using 2D phosphor-peptide mapping *in vitro* (Cdk5-p25 purified from porcine brains) and in cultured cells (co-transfected Cdk5-p35 or Cdk5-p25) and primary neurons (endogenous Cdk5-p35) using isotope labeling methods (Wada et al., 1998; Sakaue et al., 2005; Yotsumoto et al., 2009; Kimura et al., 2013). The major *in vitro* Cdk5 phosphorylation sites determined were Ser202 or Thr205 (spot 2), Ser235 (spot 4), and Ser404 (spots 3 and 5; **Figure 3A**). A similar *in vitro* 2D-phosphopeptide pattern has been reported by Illenberger et al. (1998). These sites were detected as major sites in cultured COS-7 cells when Cdk5-p25 was co-transfected and in primary neurons (**Figure 3A**; Sakaue et al., 2005). These results indicated that Cdk5 is a major kinase that phosphorylates tau in cultured neurons. The signal of spot 1, which is a doubly phosphorylation spot of Ser202 and Thr205, is strong in cultured neurons but weak when phosphorylated by Cdk5 *in vitro*. Interestingly, Ser202 and Thr205 are exclusive phosphorylation sites for Cdk5, but only one of these sites is phosphorylated, with a preference for Ser202 by Cdk5 (**Figure 3B**; Kimura et al., 2013). However, both sites can be phosphorylated by Cdk5 on the tau molecule when bound on microtubules (Wada et al., 1998). The strong signal on spot 1 in cultured neurons suggests that most tau in neurons binds to microtubules or that the sites are phosphorylated by multiple kinases.

**FIGURE 4:**
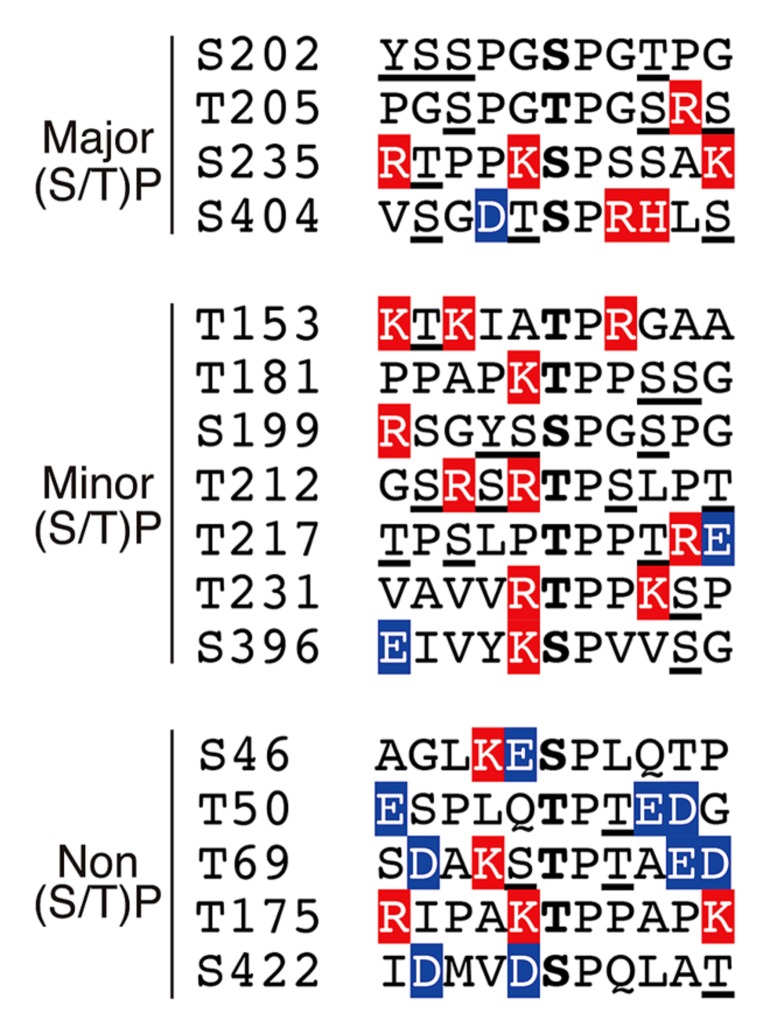
**Amino acid sequences around the (Ser/Thr)Pro motif in tau.** Ser or Thr residues N-terminal next to Pro are in the center. Basic and acidic amino acids are highlighted in red and blue, respectively. Phosphorylation sites reported thus far have been underlined.

According to the phosphorylation sites described above, the (Ser/Thr)-Pro sequences in tau can be grouped into three categories: (1) major Cdk5 phosphorylation sites, (2) minor Cdk5 phosphorylation sites and (3) non-Cdk5 phosphorylation sites (**Figure 4**). As tau is a filamentous protein, the amino acid sequence around the phosphorylation sites may affect their phosphorylation ability more than those in globular proteins. Thus, several interesting aspects can be observed; three of the four major sites, specifically Ser202, Thr205 and Ser235, have Pro at the second N-terminal site, that is, Pro-X-(Ser/Thr)-Pro, which is also a consensus sequence for MAP kinase (Davis, 1993). Thus, these sites may be commonly phosphorylated by MAP kinase. Ser404 is a nearly perfect consensus of Cdk5. Four of the six minor phosphorylation sites have Lys or Arg at the N-terminal next to the phosphorylation sites: (Lys/Arg)-(Ser/Thr)-Pro. These Lys-Ser-Pro (KSP) sequences are phosphorylation sites found in neurofilament H and M subunits (Hisanaga et al., 1993; Kesavapany et al., 2003), and phospho-specific antibodies for these sites are known to react with phospho-tau (Nukina et al., 1987; Lichtenberg-Kraag et al., 1992). In contrast to these phosphorylation sites, there are many acidic amino acids around non-Cdk5 phosphorylation sites (labeled in blue in **Figure 4**), except for Thr175. These acidic amino acids may decrease the propensity of phosphorylation. Thus, Cdk5 cannot phosphorylate the (Ser/Thr)-Pro site if it is already phosphorylated. This finding is consistent with the exclusive relationship between Ser202 and Thr205 phosphorylation and may also explain why Ser231 and Ser199 are minor phosphorylation sites, as they have a better Cdk5 phosphorylation site close to them. This is in contrast to GSK3β, which requires priming phosphorylation around the phosphorylation sites (**Figure 1**; Cohen and Frame, 2001).

The Cdk5 phosphorylation sites described above are the results of *in vitro* or cultured cell experiments. However, we believe that these properties can be applied to *in vivo* phosphorylation. As described above, Ser404 appears to be a Cdk5-specific site. Ser404 is one of the most phosphorylated sites in rat brain independent of whether the phosphorylation was examined using mass spectroscopy or an anti-phospho-antibody (Arioka et al., 1993; Watanabe et al., 1993; Yu et al., 2009), which suggest that Cdk5 is a major tau kinase in normal brains. Indeed, knockdown of Cdk5 reduced tau phosphorylation at the PHF-13 site (Ser396/Ser404; Piedrahita et al., 2010), and transgenic expression of p25 in mouse brain increases tau phosphorylation at several sites, including AT8 and PHF-1 sites (Ahlijanian et al., 2000; Cruz et al., 2003; Noble et al., 2003). In contrast, several reports argue against *in vivo* Cdk5 phosphorylation of tau. For example, phosphorylation of tau is not decreased in mice with a null mutation of p35 (Hallows et al., 2003). Furthermore, an increase in overall tau phosphorylation was not detected in p25 transgenic mice (Takashima et al., 2001; Plattner et al., 2006), although phosphorylation at specific sites such as Ser202 and Ser235 was shown to increase in another p25 mouse model (Wen et al., 2008). Tau expressed in yeast lacking the Cdk5 homolog *Pho85* showed an increase, not a decrease, in phosphorylation (Vandebroek et al., 2005). These reports demonstrate GSK3β to be the predominant tau kinase and its inhibition via Cdk5 (Hallows et al., 2003; Plattner et al., 2006; Wen et al., 2008). However, one challenge is the lack of methods used to specifically detect Cdk5 phosphorylated sites. Phospho-specific antibodies have been used. The most frequently used antibodies are AT-8 and PHF-1; however, these antibodies require multiple phosphorylation for immunoreaction; for example, for AT-8, it requires phosphorylation at Ser199 in addition to Ser202 or Thr205 phosphorylation by Cdk5; for AT180, a recent report indicates AT180 requires only Thr231 phosphorylation (Amniai et al., 2011) although it was often used as evidence for phosphorylation at both Thr231 and Ser235; and for PHF-1, it requires phosphorylation at Ser369 following Ser404 phosphorylation by Cdk5. Thus, specific tools for each Cdk5 site alone are required.

## ABNORMAL OR PATHOLOGICAL PHOSPHORYLATION OF tau BY Cdk5

It is generally considered that phosphorylation of tau by Cdk5-p35 is physiological and that phosphorylation by Cdk5-p25 is pathological. Most *in vitro* experiments have been done using Cdk5-p25 because purified Cdk5 is complexed with p25. Thus, these results should include information on abnormal phosphorylation of tau. Nevertheless, the entity of hyperphosphorylation is still unclear; is there an increase in the number of phosphorylation sites, an increase in the extent of phosphorylation at these particular sites, or both? Two biochemical studies have addressed the kinetics of tau phosphorylation using recombinant Cdk5-p35 and Cdk5-p25 (Hashiguchi et al., 2002; Peterson et al., 2010). However, both studies arrived at different conclusions. One study showed a higher affinity of Cdk5-p25 for tau compared to Cdk5-p35 (Hashiguchi et al., 2002), while the other did not obtain similar findings (Peterson et al., 2010). Considering the multiple phosphorylation sites in tau with different amino acid sequences surrounding the sites, it may be difficult to determine the kinetic parameters of tau phosphorylation *in vitro*. Cellular phosphorylation may be even more complicated and is affected by the accessibility of Cdk5 activated by p35 and p25, phosphorylation by other kinases and the binding of tau to microtubules. Our simple comparison using 2D-phospho-peptide mapping indicates that the major phosphorylation spots were similar between tau phosphorylated by Cdk5-p35 and Cdk5-p25 (Sakaue et al., 2005). On the basis of these results, we propose that Cdk5-dependent abnormal phosphorylation represents an increase in the rate of phosphorylation at the particular sites rather than an increase in the number of sites.

An increase in phosphorylation by Cdk5 would further elevate the total phosphorylation of tau by facilitating subsequent phosphorylation with GSK3β. GSK3β is another tau kinase, which is also known as TPKI (Ishiguro et al., 1992). GSK3β phosphorylates Ser/Thr residues with priming phosphorylation at the +4 site, (Ser/Thr)-X_3_-(pSer/pThr). In fact, GSK3β phosphorylation is considerably enhanced by prior phosphorylation with Cdk5 (Arioka et al., 1993; Sengupta et al., 1997; Li et al., 2006). Although many GSK3β sites have been reported (Chauhan et al., 2005; Hanger et al., 2009), the major sites are Ser199, Ser202, Thr231, Ser396, Ser400, and Ser412 (Imahori and Uchida, 1997). Thr 231 and Ser396/Se400 are primed by phosphorylation at Ser235 and Ser404 by Cdk5, respectively (Li et al., 2006). Thus, Cdk5 would increase the total phosphorylation of tau with GSK3β in an additive manner.

**FIGURE 5:**
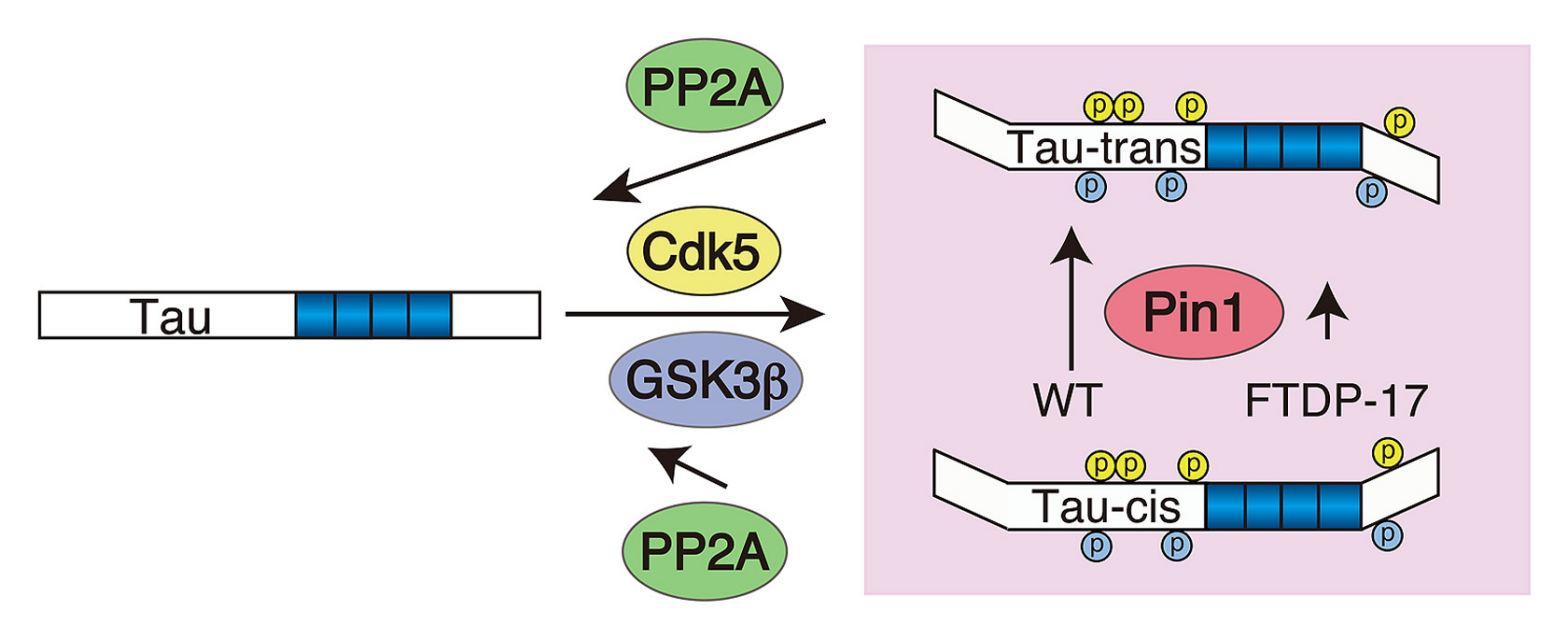
**Schematic representing the contribution of Pin1 to the hyperphosphorylation of FTDP-17 mutant tau.** Tau is phosphorylated by Cdk5 (yellow) and subsequently by GSK3β (blue) at (Ser/Thr)Pro sites. Pin1 changes the conformation of the peptide bond at p(Ser/Thr)Pro from *cis* to *trans*, thereby facilitating its dephosphorylation with PP2A. FTDP-17 mutants have a weak affinity to Pin1 compared to WT tau.

The net phosphorylation is a result of the balance between phosphorylation and dephosphorylation. Hyperphosphorylation should be attained by either increased phosphorylation or decreased dephosphorylation. What is the contribution of dephosphorylation? As previously reported (Sontag and Sontag, 2014), protein phosphatase 2A is a major phosphatase for tau. The dephosphorylation velocity of tau at Cdk5 sites is slower compared to PKA phosphorylation sites (Yotsumoto et al., 2009), which may, at least in part, be due to the stronger resistance of phosphorylation at the (Ser/Thr)Pro sites against PP2A-dependent dephosphorylation compared to other sites. Dephosphorylation at the (Ser/Thr)Pro sites is modulated by Pin1 peptidyl-prolyl *cis*/*trans* isomerase (Lu et al., 1999). Pin1 changes the conformation of the peptide bond at proline from *cis* to *trans* (Lu et al., 1996; Lu and Zhou, 2007; Driver et al., 2014), and the *trans*-conformation is easily dephosphorylated by PP2A (**Figure 5**). Dephosphorylation of Cdk5 phosphorylation sites at Ser202 and Ser235 is delayed in the absence of Pin1 (Kimura et al., 2013). The contribution of Pin1 in tau aggregate formation in AD has been demonstrated in Pin1-deficient mouse brains (Liou et al., 2003). In addition to the four major Cdk5 sites, dephosphorylation at Ser212 and Thr231 is also stimulated by Pin1 (Lu et al., 1999; Smet et al., 2004), although it is recently shown that dephosphorylation of Thr231 is not Pin1-dependent (Landrieu et al., 2011). There may be more (Ser/Thr)-Pro phosphorylation sites in which dephosphorylation is regulated by Pin1. We also demonstrated that phosphorylation of FTDP-17 mutant tau, P301L and R406W, by Cdk5 exhibits a slightly weaker affinity to Pin1 compared to WT tau, which may result in decreased dephosphorylation of mutant tau by PP2A, consistent with previous reports that the FTDP mutation demonstrates weakened binding to PP2A (Goedert et al., 2000).

## PHOSPHORYLATION OF FTDP-17 tau MUTANTS BY Cdk5

Frontotemporal dementia with parkinsonism linked to chromosome 17 is a dominantly inherited disease of neurodegenerative dementia with mutations in the tau gene *MAPT* (Hutton et al., 1998; Poorkaj et al., 1998; Spillantini et al., 1998). There are more than 40 FTDP-17 mutations reported, most of which are missense mutations found in the microtubule binding repeats with four mutations at the N-terminal and six in the C-terminal regions (**Figure 1**; Spillantini and Goedert, 2013). FTDP-17 mutant tau forms aggregates in the frontal cortex of patient brains and tau is abnormally phosphorylated at the sites of phosphorylation in AD (Crowther and Goedert, 2000; Lee et al., 2005). Thus, it is thought that these mutations are highly involved in abnormal phosphorylation. Phosphorylation of FTDP-17 mutants has been studied predominantly in mice overexpressing mutant tau. Overexpressed mutant tau forms sarkosyl-insoluble aggregates in mouse brains, and aggregates can be labeled using phospho-specific antibodies (Lewis et al., 2000; Gotz et al., 2001; Tanemura et al., 2002; Tatebayashi et al., 2002; Ikeda et al., 2005; Yoshiyama et al., 2007). However, similar to AD tau, it is not known how FTDP-17 mutation induces abnormal phosphorylation.

Several groups have examined the phosphorylation of FTDP-17 tau mutants by Cdk5. Han et al. (2009) showed that mutations of P301L, V337M, R406W, and G272V promoted the mobility shift of tau upon phosphorylation by Cdk5, whereas R406W inhibited Ser404 and Ser235 phosphorylation. They concluded that the increased mobility shift was due to an increase in phosphorylation at Ser202, Ser404, and Ser235 but not due to the higher extent of total phosphorylation. Vanhelmont et al. (2010) studied the phosphorylation of six FTDP-17 mutants, namely G272D, N279K, ΔK280, P301L, V337M, and R406W, in yeast strains lacking *mds1* GSK3β homolog and/or *Pho85* Cdk5 homolog. They demonstrated that P301L and R406W showed lower AD2 (phosphorylation at Ser396/Ser404) and PG5 (phosphorylation at Ser409) reactivity and that the reaction was reduced in the *mds1*-lacking strain and stimulated in the *Pho85*-deficient strain, suggesting that Ser396/Ser404 sites are phosphorylated by GSK3β and mutations affecting Ser409 phosphorylation. We have examined the phosphorylation of K257T, P301L, P301S, and R406W mutants *in vitro* and in cultured cells by Cdk5 using 2D-phosphopeptide mapping (Sakaue et al., 2005; Yotsumoto et al., 2009). The phospho-peptide patterns were identical between WT and mutant tau except for R406W. Phosphorylation at Ser404 was lacking in R406W. We propose that the R406W mutation alters the preferred consensus at the Ser404 site for Cdk5 to less preferred sequences. A distinct difference in phosphorylation was consistently reported with R406W tau, although these results were variable; reduced phosphorylation at many phosphorylation sites (DeTure et al., 2002), reduced phosphorylation at specific sites (Alonso Adel et al., 2004; Gauthier-Kemper et al., 2011), or greater overall phosphorylation was observed (Krishnamurthy and Johnson, 2004). One R406W patient showed a different progression of the disease from other FTDP-17 patients. Thus, it would be interesting to determine the role of this specific property of phosphorylation in disease development.

## INSULT-INDUCED PHOSPHORYLATION OF tau BY Cdk5 IN BRAINS

Numerous reports have described an increased phosphorylation of tau when neurons suffer from various neurotoxic insults, such as Aβ (Otth et al., 2002; Han et al., 2005; Zheng et al., 2005, 2010; Lopes et al., 2007; Zempel et al., 2010; Shukla et al., 2012), ischemia/hypoxia (Wen et al., 2007; Barros-Miñones et al., 2013), oxidative stress (Absalon et al., 2013), inflammation (Quintanilla et al., 2004; Kitazawa et al., 2005), and excitotoxicity (Ho et al., 2002). Aging may also represent a specific stress factor (Kelleher et al., 2007). Many of these studies observed an increase in p25 and argued Cdk5-dependent phosphorylation of tau. However, the increase in p25 and tau phosphorylation were performed in parallel and their direct correlation was not mostly demonstrated. Considering that Cdk5 sites can be phosphorylated by other PDPKs and that relatively minor phosphorylation sites were examined using western blotting analyses with anti-phospho-specific antibodies, further studies are required to determine their causal relationship.

## CONCLUSION

Elucidation of the molecular mechanism inducing hyperphosphorylation of tau in tauopathic brains including AD is one of the critical issues for the prevention of dementia development independent of hyperphosphorylation as a cause of disease. Hyperphosphorylation must reflect the cellular conditions of affected neurons in disease brains. Cdk5 has been extensively studied as one of the major kinases because Cdk5 generates disease-specific phosphorylation epitopes. However, despite intensive previous studies, it is still unclear how Cdk5 contributes to tau phosphorylation physiologically and pathologically. In particular, *in vivo* phosphorylation by Cdk5 has not been convincingly demonstrated. This may, at least in part, be due to an overlap in the phosphorylation of many (Ser/Thr)Pro sites in tau by several PDPKs. Another possibility is the lack of methods used to specifically identify Cdk5 phosphorylation. By overcoming these challenges, studies on tau phosphorylation by Cdk5 can provide valuable insight on the molecular mechanism underlying AD and the development of strategies to prevent dementia.

## Conflict of Interest Statement

The authors declare that the research was conducted in the absence of any commercial or financial relationships that could be construed as a potential conflict of interest.
